# Correction: Hybrid optimal-FOPID based UPQC for reducing harmonics and compensate load power in renewable energy sources grid connected system

**DOI:** 10.1371/journal.pone.0305301

**Published:** 2024-06-06

**Authors:** T. Anuradha Devi, G. Srinivasa Rao, T. Anil Kumar, B. Srikanth Goud, Ch. Rami Reddy, Mbadjoun Wapet Daniel Eutyche, Flah Aymen, Claude Ziad El-Bayeh, Habib Kraiem, Vojtech Blazek

The eighth author’s name is spelled incorrectly. The correct name is: Claude Ziad El-Bayeh.

## References

[1] Devi TA, Rao GS, Kumar TA, Goud BS, Rami Reddy C, Eutyche MWD, et al. (2024) Hybrid optimal-FOPID based UPQC for reducing harmonics and compensate load power in renewable energy sources grid connected system. PLoS ONE 19(5): e0300145. 10.1371/journal.pone.0300145 38743740 PMC11093348

