# Supplementary material for: Hybrid optimal-FOPID based UPQC for reducing harmonics and compensate load power in renewable energy sources grid connected system
Source: PLoS One. 2024 May 14;19(5):e0300145. doi: 10.1371/journal.pone.0300145 (PMC11093348; doi:10.1371/journal.pone.0300145)
Supplement: Attachment — Submitted filename: LOR.docx [file pone.0300145.s001.docx]

I COULD RECOMMEND THIS MANUSCRIPT FOR ACCEPTANCE IN ACCORDANCE WITH THE REVISIONS CARRIED OUT AND THE DETAILED DESCRIPTION OF THE CORRECTIONS CARRIED OUT.
