# Supplementary material for: Hybrid optimal-FOPID based UPQC for reducing harmonics and compensate load power in renewable energy sources grid connected system
Source: PLoS One. 2024 May 14;19(5):e0300145. doi: 10.1371/journal.pone.0300145 (PMC11093348; doi:10.1371/journal.pone.0300145)
Supplement: Attachment — Submitted filename: Recommendation.docx [file pone.0300145.s002.docx]

Language used in the article needs to be refined.

“The pulse signal of UPQC was done using the optimal controller, which analyzes the error value of reference value and actual value to generate pulses.” Meaning is not clear

“The most cutting-edge renewable energy sources among them are PV and WECS, which are frequently used in all systems [3]” – What is WECS and how are these natural sources classified as cutting edge?

What is the parameter given in Eq 1. Why is it needed in the study?

“. That is Coati Optimization Algorithm (COA) is utilized for the initialization process and the Osprey Optimization Algorithm (OOA) is utilized for the initialization process.” – needs explanation

Units missing in Fig.3.

Figs 22, 24, 28, 21, 19, 17, 12, 10, 8, 33, 35, 37 , 40, 42, 44 axes labels and titles not clear. Need to change.

Explanation of Table 3 needed.

Please compare the PF and THD of the proposed system to industry standards.
