# Supplementary material for: Hybrid optimal-FOPID based UPQC for reducing harmonics and compensate load power in renewable energy sources grid connected system
Source: PLoS One. 2024 May 14;19(5):e0300145. doi: 10.1371/journal.pone.0300145 (PMC11093348; doi:10.1371/journal.pone.0300145)
Supplement: Attachment — Submitted filename: Reviewer 4.docx [file pone.0300145.s003.docx]

**Reviewer 4**

The idea is good written but it needs further improvements like:

1. The contribution is not illustrated in an introduction. Make it in points.

Response: We thank the reviewer for the suggestion made. We have now included the contribution of the work in bullet point after the introduction section as below

- A novel hybrid optimization-based FOPID controller was developed to improve PQ in integrated microgrid systems in order to mitigate these problems. That is both the Coati Optimization Algorithm (COA) and Osprey Optimization Algorithm (OOA) are combined to make a hybrid optimization CO-OA algorithm. Hybrid optimization reduces the time of the decision-making process and allows to focus their time on the analyses.
- Designed to increase PQ in a hybrid RES-grid connected non-linear distribution system. A hybrid RES based Grid with a load model was designed as per specific ranges, in between load and hybrid RES an UPQC is linked for PQ management.
- The pulse generation of UPQC compensator's switches is done through the use of the FOPID controller, which compares the actual value and reference value to generate the pulse signal of the switches. FOPID controller's parameters are tuned by using an innovative hybrid optimization algorithm.
- A low error function is used to choose the best optimal values. The proposed mitigation performance is analyzed under sag, harmonics, interruption, and swell conditions. Moreover, the outcomes are compared to some other existing models to confirm the efficacy of the proposed model.

2. Implementation of optimization techniques utilized is not validated. Explain in detail how to implement your model with optimization.

Response: We thank the reviewer for the following comment. We have validated the Controller gains/parameter in the Table.

Hybrid optimization parameters

| Parameter | Ranges |
| --- | --- |
| Iteration | 100 |
| Dimension | 5 |
| T | 0.01 |
| Search agent | 30 |

Table. 1 Controller parameters I mean gains

| **Parameter** | **Ranges** |
| --- | --- |
| µ | 0.0455 |
| K_D_ | 0.0427 |
| K_I_ | 2.9161 |
| K_P_ | 0.0284 |
| λ | 0.0313 |

3. Make a deeply comparative analysis between different controllers and different optimization techniques in terms of objective function, control parameters, and computation times.

Response: We thank the reviewer for the suggestion made. The following figures clearly shows the comparison of convergence in proposed and existing algorithms like the Aquila Optimization Algorithm (AOA), Pelican Optimization Algorithm (POA), African Vulture Optimization (AVO) and Ant Lion Optimization (ALO) Algorithm. If the relevant sequence converges for specified initial approximations, an iterative process is said to be convergent.

Figure 46: Analysis of convergence comparison

In the proposed hybrid algorithm, the 18th iteration to overcome the error is 130. In existing methods, AOA attains the 10th error state in the 7th iteration, POA attains the 40th error state in the 5th iteration, AVO attains the 35th error state in the 10th iteration and ALO attains the 9th error in the 15th iteration. Compared to existing methods, the proposed method effectively performs.

4. Articles may be used in literature like:

10.1109/ACCESS.2023.3317980
10.3390/axioms12050420
10.3390/su15065209
10.3390/su15043710

Response: we thank the reviewer for sharing the recent papers in the same area of the manuscript which added more inputs in revising the manuscript
